# The Association Between Diabetes Mellitus and Atrial Fibrillation: Clinical and Mechanistic Insights

**DOI:** 10.3389/fphys.2019.00135

**Published:** 2019-02-26

**Authors:** Loryn J. Bohne, Dustin Johnson, Robert A. Rose, Stephen B. Wilton, Anne M. Gillis

**Affiliations:** Department of Cardiac Sciences and Department of Physiology and Pharmacology, University of Calgary and Libin Cardiovascular Institute of Alberta, Calgary, AB, Canada

**Keywords:** atrial fibrillation, diabetes mellitus, risk factors, mechanisms, atrial remodeling

## Abstract

A number of clinical studies have reported that diabetes mellitus (DM) is an independent risk factor for Atrial fibrillation (AF). After adjustment for other known risk factors including age, sex, and cardiovascular risk factors, DM remains a significant if modest risk factor for development of AF. The mechanisms underlying the increased susceptibility to AF in DM are incompletely understood, but are thought to involve electrical, structural, and autonomic remodeling in the atria. Electrical remodeling in DM may involve alterations in gap junction function that affect atrial conduction velocity due to changes in expression or localization of connexins. Electrical remodeling can also occur due to changes in atrial action potential morphology in association with changes in ionic currents, such as sodium or potassium currents, that can affect conduction velocity or susceptibility to triggered activity. Structural remodeling in DM results in atrial fibrosis, which can alter conduction patterns and susceptibility to re-entry in the atria. In addition, increases in atrial adipose tissue, especially in Type II DM, can lead to disruptions in atrial conduction velocity or conduction patterns that may affect arrhythmogenesis. Whether the insulin resistance in type II DM activates unique intracellular signaling pathways independent of obesity requires further investigation. In addition, the relationship between incident AF and glycemic control requires further study.

## Introduction

Atrial fibrillation (AF) is the most common sustained arrhythmia and is associated with substantial morbidity and mortality. Risk factors for atrial fibrillation include age, hypertension, obesity, valvular heart disease, heart failure, and obstructive sleep apnea (Benjamin et al., 1994; Staerk et al., 2017). Some but not all studies have reported that diabetes mellitus (DM) is an independent risk factor for AF (Benjamin et al., 1994; Krahn et al., 1995; Huxley et al., 2011). Here we will review the clinical data supporting the association between DM and AF and discuss the potential mechanism(s) by which DM may contribute to the electrophysiologic substrate for AF. We also suggest future potential research studies to advance our knowledge in this field.

## Association Between DM and AF: Clinical Studies

A potential association between DM and incidence of AF has long been postulated based on epidemiologic studies. The original Framingham Heart Study consisted of 5,209 patients (2,336 men, 2,873 women), age 30–62 years of age. This study initiated in 1948 represented a random sample of two thirds of the population of Framingham, Massachusetts and has conducted pivotal research defining cardiovascular risk factors (Tsao and Vasan, 2015). This study reported that hypertension, diabetes, congestive heart failure and valvular heart disease were independent risk factors for AF in both men and women (Benjamin et al., 1994). The odds ratio (OR) of risk of developing AF in the association with DM was 1.4 (95% CI 1.0–2.0) and 1.6 (95% CI 1.1–2.2) for men and women, respectively. However, in the absence of valvular heart disease, diabetes was no longer a significant risk predictor for the development of AF. The Framingham Heart Study did not include body mass index (BMI) or a history of obstructive sleep apnea in this initial analysis.

The Manitoba Follow-Up Study which also commenced in 1948 prospectively followed 3,983 healthy male air crew recruits for 44 years. The goal of this study was to examine the role that abnormalities on the resting electrocardiogram might play in the prediction of future cardiovascular disease. In this cohort, obesity [relative risk (RR) 1.28; 95% CI 1.02–1.62] but not DM was reported to be an independent risk factor for AF (Krahn et al., 1995).

In 2011, Huxley et al. reported the results of a systematic review and meta-analysis examining the association between DM and AF (Huxley et al., 2011). They identified seven prospective cohort studies and four case control studies that included 108,703 individuals with AF and 1,686,097 control subjects. Overall, DM was associated with a 39% increased risk of AF compared to controls (RR 1.39, 95% CI 1.10–1.75). However, only three of these studies reported multivariable adjusted risk estimates for AF (adjusting for age and some other risk factors). Perhaps, not surprisingly, after adjusting for these known factors the association between DM and AF was significantly attenuated and in two of these studies was no longer statistically significant. Thus, the association between DM and incident AF has likely been overestimated in this meta-analysis. Furthermore, these previous studies did not specifically distinguish between Type I or Type II DM, glycemic control or specific therapies.

**Figure 1:**
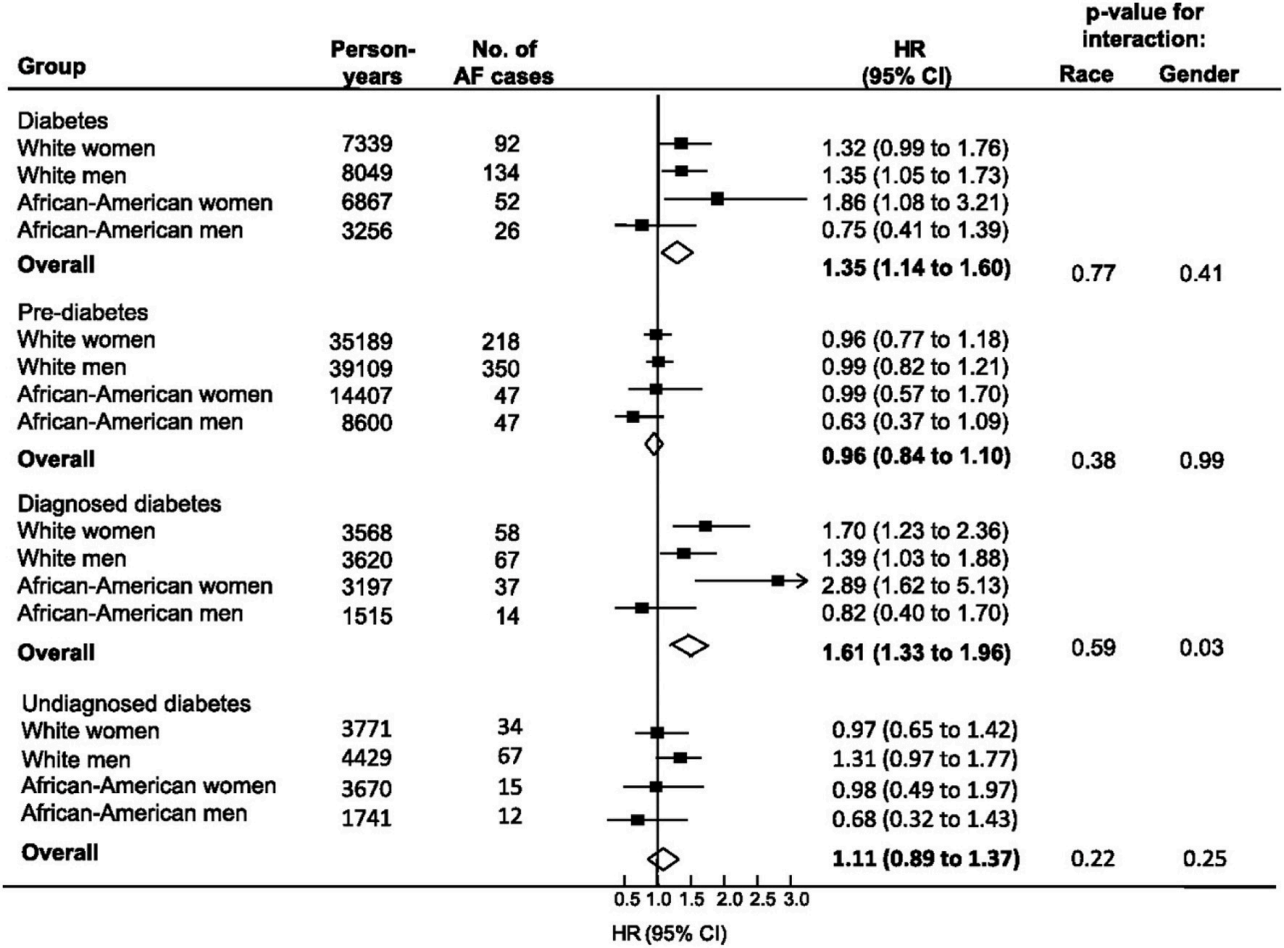
Relationship between diabetes, pre-diabetes, and physician-diagnosed diabetes with incident atrial fibrillation from the ARIC study (1990–2007). Individuals without diabetes comprised the reference group for each comparison. Diabetes included all individuals with FSG > 126 mg/dl or HbA1c > 6.5% or use of diabetic medication or history of physician-diagnosed diabetes. Undiagnosed diabetes defined as fasting serum glucose (FSG) > 126 mg/dl or HbA1c > 6.5% but no history of diabetic medication usage or physician diagnosed diabetes. Black boxes represent the estimate adjusted for age, education, income, prior history of cardiovascular disease, BMI, systolic blood pressure, use of hypertensive medications, and smoking. Horizontal lines represent 95% confidence intervals. Open diamonds represent the estimate of effect for the overall population. Note the strong correlation between DM and AF among African American women. Reproduced with permission from Huxley et al. (2012).

Since this meta-analysis was published, several additional observational studies examining the association between DM and AF have been reported. The Atherosclerosis Risk in Communities study is comprised of 15,792 white and African American patients age 45–64 years pooled from 4 geographically distinct communities in the United States. A 2012 report from this cohort examined the associations between Type II DM, markers of glucose homeostasis and risk of AF (Huxley et al., 2012). After adjustment for a number of risk factors, DM was associated with a significant increased risk for AF [hazard ratio (HR) 1.35, 95% CI 1.14–1.60] (Figure 1). However, those with pre diabetes or undiagnosed diabetes were not at increased risk for AF. The authors also reported a positive linear association between HbA1c and the risk of AF for both those with and without DM, though a similar positive relationship between fasting blood glucose and risk for AF was observed only in patients with previously diagnosed DM (Huxley et al., 2012).

In the Women's Health Study, Type II DM was a significant predictor of risk for AF (HR 1.37; 95% CI 1.03–1.83) after adjustment for cardiovascular risk factors including hypertension and BMI (Schoen et al., 2012). However, these investigators observed that the development of hypertension, obesity, and cardiovascular disease over time were stronger predictors of risk of incident AF compared to DM. In a subgroup analysis of the Women's Health Study, baseline HbA1c was not predictive of subsequent AF.

Risk factors for AF were evaluated in post-menopausal women in the Women's Health Initiative Observational Study (Perez et al., 2013). Consistent with earlier observational cohort studies, age, hypertension, obesity, diabetes, myocardial infarction, and congestive heart failure were all independently associated with incident AF. However, hypertension and overweight status/obesity contributed to 28.3 and 12.1% of the population attributable risk for AF whereas DM accounted for only 3.4% of this risk. A Korean National Health Insurance Service Database analysis confirmed that after adjustment for age, sex, BMI, and other covariates the population attributable risk of AF for hypertension, ischemic heart disease, heart failure and DM were 16, 8.2, 5.3, and 0.8%, respectively (Son et al., 2016). In this latter analysis, DM was not a significant risk predictor for AF.

A Danish Nation cohort study evaluated the risk of AF in individuals with DM compared to the background Danish population (Pallisgaard et al., 2016). After adjustment for multiple covariates, DM was associated with a relative 19% increased risk of incident AF. Interestingly, the incidence rate ratio was highest in the youngest age group 18–39 years (2.34, 95% CI 1.52–3.60) compared to older patients (1.20, 95% CI 1.18–1.23 age group 65–74). This analysis did not stratify relative risks for those with Type I vs. Type II DM or describe the duration of DM prior to AF diagnosis, so the apparent effect modification by age is not easily explained. The relative decrease in risk of AF in DM with increasing age may reflect the importance of other risk factors for AF that occur with advancing age.

**Figure 2:**
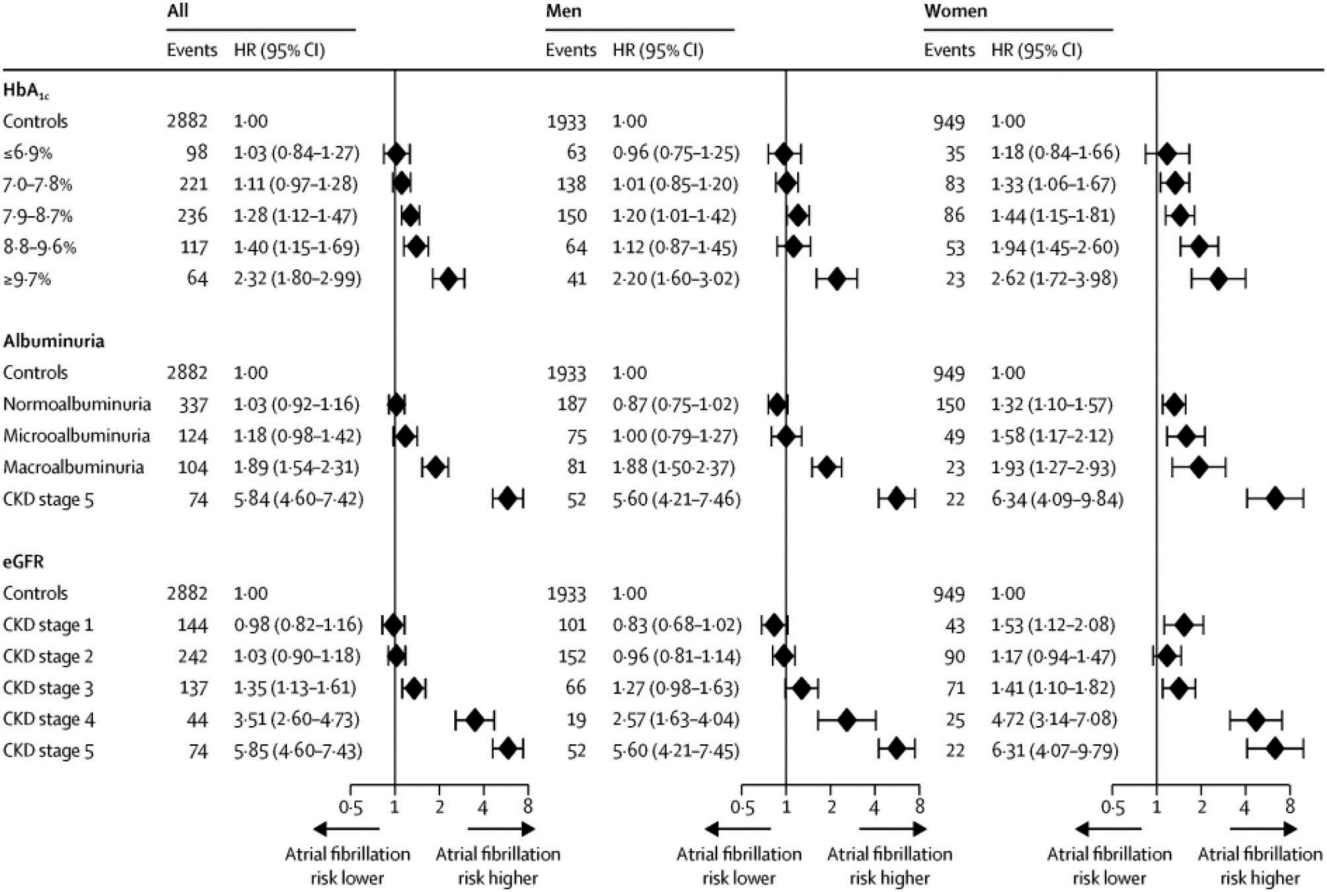
Risk of AF in Type I DM vs. controls based on HbA_1c_ category, albuminuria category, and chronic kidney disease (CKD) stage. CKD stages 1–4 are based on eGFR (estimated glomerular filtration rate): CKD stage 1—eGFR ≥ 90 mL/min per 1.73 m^2^; CKD stage 2—eGFR 60–89 mL/min per 1.73 m^2^; CKD stage 3—eGFR 30–59 mL/min per 1.73 m ^2^; CKD stage 4—eGFR 15–29 mL/min per 1.73 m^2^;CKD stage 5—eGFR < 15 mL/min per 1.73 m^2^ or need for dialysis or kidney transplantation. Diamonds indicate HRs and error bars the 95% CIs. Reproduced with permission from Dahlqvist et al. (2017).

A recent prospective, case control study examined the relationship between Type I diabetes and AF (Dahlqvist et al., 2017). Using a Swedish database, Dahlqvist compared 36 258 patients with Type I DM to 179,980 controls followed for a median of 9.7 years. The mean age of participants was 35 years. Type I DM was associated with a modest increase in the risk of AF in men (HR 1.13, 95% CI 1.01–1.25), but a 50% increase risk of AF in women (HR 1.5, 95% CI 1.3–1.72). The risk of AF was increased in those with worse glycemic control and renal complications (Figure 2).

In this issue of Frontiers in Physiology (Xiong et al., 2018), have summarized the current state of the literature in an updated systematic review and meta-analysis including publications reported up to September 2017 that investigated the association between DM and AF. They used a novel machine learning approach to identify publications suitable for analysis. Twenty-nine studies (21 observational cohort or randomized trials and 8 case control studies) including 8,037,756 subjects were included in the analysis. Overall DM was associated with a pooled 49% increased risk of developing AF (RR 1.49, 95% CI1.24–1.79). However, many studies did not adjust for known risk predictors including age, sex and cardiovascular risk factors. When restricted to studies reporting adjusted estimates, DM was associated with a weaker but still significant increased risk of new onset AF (RR 1.23, 95 % CI 1.03–1.46). Furthermore, there was significant between-study heterogeneity in the observed associations that could not be explained by available differences in baseline covariates, type of DM, or study era. Interestingly, in this study the association between DM and incident AF was stronger in women than in men (RR 1.38, 95% CI 1.19–1.60 vs. 1.11, 95% CI 1.01–1.22). In addition, the association between DM and incident AF was stronger in more recent vs. older studies.

The clinical studies discussed are summarized in Table 1. These clinical studies examining the potential association between DM and AF incidence share important limitations. Most publications are secondary reports from large cohorts assembled for another purpose, without detailed characterization of the type, duration or severity of DM, nor standardized AF detection protocols. Furthermore, there has been variable adjustment for confounding by other risk factors such as obesity, obstructive sleep apnea, and heart failure. This may in part explain the contradictory results.

**Table 1:** Characteristics of Clinical Studies Examining the Relationship Between Diabetes Mellitus and Atrial Fibrillation.

**Authors**	**Study design**	**Findings**	**Limitations**	**Diabetes type**
Benjamin et al., 1994	Prospective cohort 2,090 men 2,641 women Ages 55–94 years	HTN, DM, CHF, VHD independent risk factors for AF. DM risk: OR 1.4 men, 1.6 women	DM not a risk factor for AF after controlling for valvular heart disease. BMI and OSA not included in analysis	Not specified
Krahn et al., 1995	Prospective cohort 3,983 men	Abnormalities in resting ECG did not predict AF. Obesity but not DM a risk for AF		Not specified
Huxley et al., 2011	Systematic review and meta-analysis of 7 prospective cohort studies, 4 case control studies. 108,703 with AF 1,686,099 control subjects	39% increased risk of AF in DM compared to controls	Effect significantly attenuated after multivariable adjusted risk estimates	Type II DM
Huxley et al., 2012	Prospective cohort 13,025 subjects	35% increased risk of AF in DM after adjustment for known risk factors	Cases of AF ascertained through hospital discharge codes	Type II DM
Schoen et al., 2012	34,720 female health professionals	37% increased risk of for AF in DM. HTN, CVD, obesity stronger predictors of AF than DM	Possible under diagnosis of DM given lack of systemic screening	Type II DM
Perez et al., 2013	Observational cohort 81,892 post-menopausal women	Age, HTN, obesity, DM, MI, CHF risk factors for AF. DM risk: HR 1.55 in multivariable analysis	98% of incident AF based on hospital discharge codes	Not specified
Son et al., 2016	Korean National Health Insurance Data 206,013 subjects age >30 years	Diabetes not a risk factor for AF	Incident AF based on disease codes	Not specified
Pallisgaard et al., 2016	Danish National Registries 5,0081,087 subjects >18 years	19% increased risk of AF in DM IRR 2.34 age 18–39 IRR 1.20 age 65–74	Incident AF based on disease codes	Not specified
Xiong et al., 2018	Systemic review and meta-analysis 21 observational cohort or randomized trials and 8 case control studies 8,037,756 subjects	RR for AF in DM 1.38 (women) 1.11 (men)	Significant between study heterogeneity in observed associations	Majority Type II DM
Dahlqvist et al., 2017	Prospective case control study 36,258 patients Type I DM 179,980 control subjects	Modest 13% increased risk of AF in men Significant 50% increased risk of AF in women. Risk greater in those with poor glycemic control	No data on blood pressure or BMI	Type I DM

*AF, atrial fibrillation; HTN, hypertension; CHF, congestive heart failure; IRR, incident risk ratio; CVD, cardiovascular disease; OR, odds ratio; DM, diabetes mellitus; RR, relative risk; HR, hazard ratio; VHD, valvular heart disease*.

Notwithstanding these limitations, DM appears to be a significant if modest risk factor for development of AF. This association may be increasing in strength over time, as secular trends in other risk factors especially better control of hypertension and management of heart failure (Schnabel et al., 2015) and an increasing incidence of obesity and DM worldwide have been reported. These changes suggest that more attention to the diagnosis and management of DM as well as aggressive weight loss interventions for overweight/obese individuals may be important in curbing the emerging epidemic of AF. The contradictory results from the available clinical data set also serves to highlight the importance of fundamental research to elucidate the mechanisms by which DM might promote AF, summarized in the next section.

## Mechanisms of AF in DM: Insights from Experimental Studies

### Mechanisms of Atrial Fibrillation

**Figure 3:**
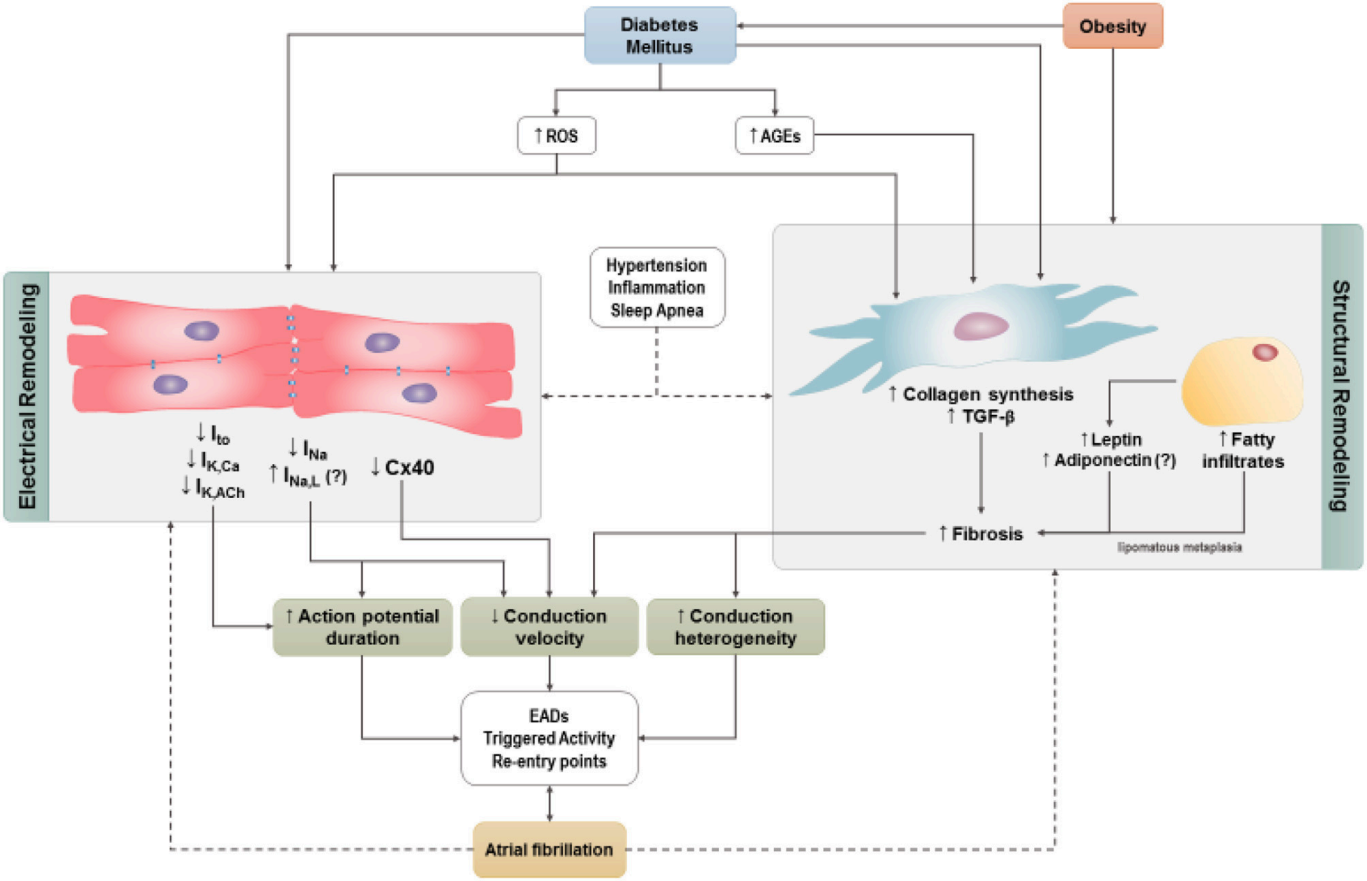
Cellular mechanisms by which diabetes may predispose to AF. Increases in reactive oxygen species and/or advanced glycation-end products trigger atrial electrical and structural remodeling. Obesity independent of DM contributes to atrial structural remodeling. Hypertension, obstructive sleep apnea, and systemic inflammation are frequently associated with DM and contribute to atrial electrical and structural remodeling. ROS, reactive oxygen species; AGE, advanced glycation-end products (AGES); I_to_, transient outward current; IK_Ca_, Ca^2+^–activated potassium channels; I_KACh_, Acetylcholine dependent potassium current; I_Na_, sodium current; I_NaL_, late inward sodium current; TGF-β, transforming growth factor-β; EADs , early after depolarizations; Cx40, connexin 40.

While the mechanisms underlying AF are incompletely understood, research efforts have identified a substantial number of pathophysiological determinants, including at the cellular and molecular levels, that can lead to electrical and structural remodeling and thereby favor the occurrence of AF. These include a number of mechanisms that can lead to triggers for AF initiation as well as mechanisms that create a substrate for AF maintenance and progression (Heijman et al., 2014, 2018). Below, we focus our discussion on alterations that have been shown or proposed to lead to AF specifically in the setting of DM. These potential mechanism(s) are outlined in Figure 3.

### Animal Models of Diabetes Mellitus and AF Research

A number of animal models of DM have been employed to study the atrial electrical and structural changes and the underlying molecular mechanism(s) that predispose to AF in this setting. These include models of Type I DM induced by chemical induction using streptozotocin or alloxan (King, 2012; Watanabe et al., 2012; Liu et al., 2014, 2017; Saito et al., 2014; Fu et al., 2015; Yi et al., 2015; Hayami et al., 2016), which have been successfully employed in rodents and rabbits. The Akita mouse model of Type 1 DM which arose due to a mutation in the insulin-2 gene replicates many of the complications of diabetes including retinopathy, neuropathy, nephropathy and increased oxidative stress (Hsueh et al., 2007). Most current animal models of type II DM are associated with obesity. These include monogenic models of leptin deficiency (mice) or deficiency in the leptin receptor (mice, Zucker fatty rats and diabetic fatty rats (King, 2012; Linz et al., 2016; Fukui et al., 2017). Type II DM induced by high fat feeding has been used in rabbits (Zarzoso et al., 2014). Models of metabolic syndrome characterized by central obesity, hyperlipidemia, glucose intolerance and hypertension have been induced using a combination of high fat and high sucrose diets in rodents (Hohl et al., 2017) and rabbits (Arias-Mutis et al., 2017).

### Atrial Structural Remodeling

Diabetes is known to exacerbate interstitial fibrosis in the atria. This is seen in both animal (Kato et al., 2008; Watanabe et al., 2012; Li et al., 2016) and human (Lamberts et al., 2014) studies. Atrial fibrosis has been demonstrated in studies of Type I DM (Kato et al., 2008) and Type II DM (Li et al., 2016). Obesity often leads to Type II DM and is associated with lipomatous metaplasia of the heart, a process that involves the transformation of fatty infiltrates into fibrotic tissue (Samanta et al., 2016). A sheep model of obesity induced by high caloric diet in the absence of DM induces significant atrial structural and electrical remodeling. The changes reported included left atrial enlargement, biatrial conduction abnormalities, increased expression of profibrotic mediators, interstitial atrial fibrosis, and an increased propensity for inducible and spontaneous AF (Abed et al., 2013; Mahajan et al., 2015). Therefore, in the case of Type II DM, it is unclear whether the observed atrial interstitial fibrosis and associated atrial conduction abnormalities are the result of either chronic diabetes or excessive adiposity alone.

#### Fibrosis

**Figure 4:**
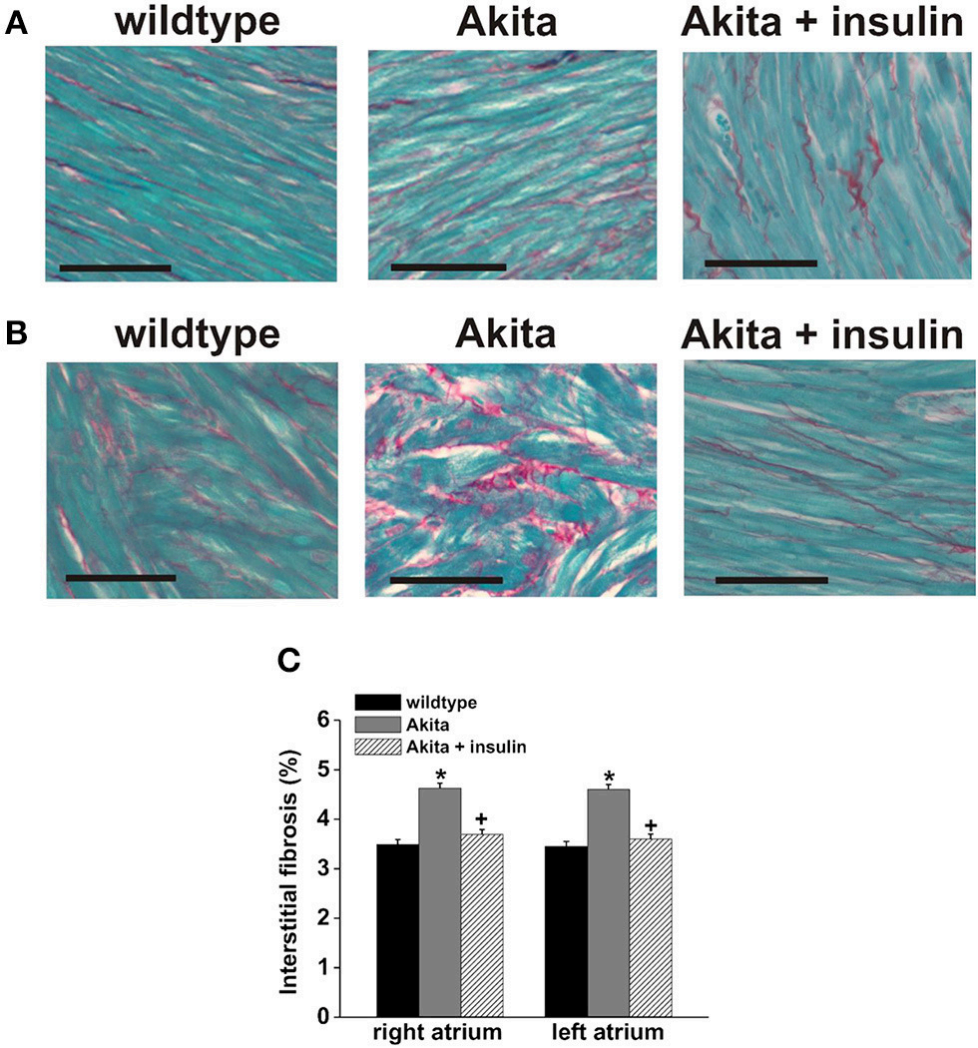
Patterns of interstitial fibrosis in the right **(A)** and left atria **(B)** of wildtype Akita mice. The myocardium is stained in green and collagen deposition in red. Scale bars are 50 μM. **(C)** The percent interstitial fibrosis is significantly increased both atria in the diabetic mice. Insulin treatment prevents the development of fibrosis in the diabetic mice, ^*^p < 0.05. Reproduced with permission from Krishnaswamy et al. (2015).

Fibrosis can be a critical contributor to the establishment of a substrate for AF as increased collagen deposition in the atria can slow atrial conduction velocity and cause fragmentation of propagating wavefronts, which can result in re-entry (Schotten et al., 2016). Fibrosis is importantly determined by the function of cardiac fibroblasts, which play a central role in the deposition of the extracellular matrix. In a number of pathological conditions, fibroblasts are activated leading to inappropriate collagen production and deposition. Cardiac fibrosis has been clearly demonstrated in diabetic patients and, consistent with this, cardiac fibroblasts isolated from the atria of patients with Type II DM show enhanced levels of collagen synthesis, as indicated by increased expression of Type I collagen (Sedgwick et al., 2014). Similar results have been observed in db/db mice, in which cultured ventricular fibroblasts showed increased expression of Type I collagen and transforming growth factor β (TGF-β) (Hutchinson et al., 2013). Since this latter study was performed in ventricular fibroblasts it remains to be determined if similar changes occur in the atria of Type II diabetic mice. On the other hand, the notion of enhanced atrial fibrosis in association with increased expression of collagens is supported in studies of Type I diabetic Akita mice in which right and left atrial fibrosis was increased in association with enhanced atrial expression of Type I and Type III collagens (Krishnaswamy et al., 2015) (Figure 4). Interestingly, the increase in atrial fibrosis in Akita mice was preventable by chronic insulin treatment.

Hyperglycemia in Type I and Type II DM is associated with enhanced angiotensin II, TGF-β signaling, and increased reactive-oxygen species (ROS) production (Singh et al., 2008a,b; Patel et al., 2012; Fiaschi et al., 2014). These are all well-characterized pro-fibrotic signaling molecules that enhance collagen synthesis and secretion by cardiac fibroblasts suggesting that these factors may contribute to atrial fibrosis and enhanced susceptibility to AF in DM. Consistent with this, angiotensin-converting enzyme inhibitors have been shown to reduce collagen and TGF-β levels in both Type I DM (Singh et al., 2008b) and Type II DM (Toblli et al., 2005). Angiotensin II is well-known to induce cardiac fibrosis and the findings mentioned above are consistent with the hypothesis that Angiotensin II is an important mediator of atrial fibrosis in DM.

Additionally, elevations in blood glucose levels stimulate the production of advanced glycation-end products (AGEs), which can enhance interstitial fibrosis by forming crosslinks between collagen and laminin (Russo and Frangogiannis, 2016). AGEs function by activating their receptors (RAGEs) located on the surface of cardiac fibroblasts, thereby upregulating connective tissue growth factor and stimulating fibroblast proliferation (Kato et al., 2008). This is referred to as the AGE-RAGE system and is thought to be another important mediator of profibrotic signaling in the atria in DM.

Atrial fibrosis, and hence the substrate for AF, may also be affected by adipokines—important signaling molecules than can be produced in the epicardial fat layer on the surface of the heart and which can act in a paracrine manner. Adipokines such as leptin and adiponectin have been implicated in atrial fibrosis. Leptin levels are elevated in obesity and DM (Karmazyn et al., 2008) and it has been demonstrated that leptin plays an important role in the development of atrial fibrosis. Specifically, the development of atrial fibrosis and the increased susceptibility to AF in response to Angiotensin II treatment increased leptin and was attenuated in leptin deficient ob/ob mice. Angiotensin II was shown to increase leptin expression in wildtype atrial fibroblasts and the addition of leptin increased TGF-β signaling (Fukui et al., 2013). A subsequent study demonstrated that a high fat diet in wildtype mice resulted in hyperleptinemia as well as high susceptibility to AF in association with increased left atrial interstitial fibrosis and that these effects were attenuated in leptin deficient ob/ob mice (Fukui et al., 2017). The role of adiponectin in AF associated with diabetes is much less clear. Adiponectin has insulin sensitizing properties and anti-inflammatory properties and the levels of adiponectin decrease with increasing adiposity (Karmazyn et al., 2008). Interestingly, higher circulating levels of adiponectin have been associated with increased risk of AF (Macheret et al., 2015). The basis for this observation is unclear and more work is needed to understand the role of adiponectin in cardiovascular diseases including diabetes and its links to AF.

#### Adipose Tissue

Type II DM presents a unique challenge in understanding the pathogenesis of AF because it typically coincides with obesity. Complications that arise from either condition are not mutually exclusive. Obesity is associated with increased thickness of epicardial adipose tissue (the fat that lies directly adjacent to the epicardium underneath the pericardium), which can have profound consequences on atrial electrophysiology and promote arrhythmogenesis (Abed et al., 2013; Mahajan et al., 2015; Evin et al., 2016). Indeed, increases in epicardial adipose tissue have been found to associate with adverse left atrial remodeling and increased incidence of AF, supporting the idea that epicardial adipose tissue could play an important role in the pathophysiology of AF (Sanghai et al., 2018). Coinciding with the increased epicardial adipose tissue volume, fatty infiltration of the atrial epicardium is also increased (Mahajan et al., 2015). Epicardial adipose tissue infiltration is associated with increased risk of AF due in part to pathological remodeling of epicardial adipose tissue itself whereby organized adipose tissue is replaced with fibro-fatty infiltrates (i.e., lipomatous metaplasia) leading to interstitial fibrosis. Indeed, an ovine model of induced AF revealed increased adipose tissue volume in the left and right atria as well as fibrosis of fatty infiltrates in comparison to their non-AF-induced counterparts (Haemers et al., 2017). Ultimately, this process impairs conduction velocity as well as homogeneity. Much like collagen fibers, fatty tissue is neither conductive nor contractile and can create physical barriers between cardiomyocytes, limiting both electro- and mechanotransduction. Additionally, the presence of these lipid deposits within the myocardium could potentially create re-entry points, further potentiating AF substrate development. It is not known if diabetes exacerbates this pathological process. Finally, epicardial adipose tissue is well-known to produce and release a number of compounds (including cytokines/adipokines, as discussed above) that could act in a paracrine fashion in the atria, thereby influencing atrial remodeling and arrhythmogenesis (Hatem and Sanders, 2014; Nagy et al., 2017). The role of paracrine effects of epicardial adipose tissue in the pathogenesis of AF is an important area for ongoing study.

### Electrical Remodeling

#### Ion Channels

Electrical remodeling in DM can also involve alterations in atrial action potential morphology due to changes in a number of ionic currents. Prolongation of the atrial action potential duration has been reported in rabbit and mouse models of Type I DM (Yi et al., 2015; Fu et al., 2016). Similar alterations have been described in the left atrium of Type II diabetic rats (Li et al., 2016). Although incompletely understood, alterations in atrial action potential morphology may involve Na^+^ and K^+^ channels, as well as Ca^2+^ homeostasis.

Evidence suggest that the Na^+^ current (I_Na_) is reduced in the atria of Type I diabetic rabbits (Liu et al., 2017). In contrast, a study in Type II diabetic rats reported no differences in atrial I_Na_ (Li et al., 2016). Accordingly, more work is needed to determine how atrial I_Na_ is affected in type I and Type II DM. This is of importance as changes in I_Na_ amplitude affect atrial action potential upstroke velocity and hence conduction velocity. In this way, I_Na_ is an important determinant of the substrate for re-entry. Another aspect of I_Na_ that may be important is the late I_Na_ (I_Na,L_) that can affect arrhythmogenesis by modulating action potential duration and the occurrence of EADs (Sicouri et al., 2013). I_Na,L_ has been shown to be increased in ventricular myocytes in Akita and db/db mice (Lu et al., 2013); however, it is presently unknown if this is also the case in the atria.

K^+^ currents play an essential role in action potential repolarization thereby controlling action potential duration and susceptibility to triggered activity (EADs). Several studies have identified changes in K^+^ currents in the atria in DM. For example, Type I diabetic (Akita) mice have been shown to have a reduction in atrial acetylcholine-activated K^+^ current (I_KACh_) in association with reduced expression of GIRK1 and GIRK4 (Park et al., 2009; Zhang et al., 2014). These alterations in I_KACh_ and GIRK expression were associated with altered insulin signaling through glycogen synthase kinase 3 and altered transcriptional regulation by sterol regulatory element binding protein 1. A reduction in I_KACh_ could affect atrial action potential duration and arrhythmogenesis; however, the links between I_KACh_ and AF have not been explored in detail.

Separate studies have shown that expression of several other K^+^ channels is altered in DM. Specifically, expression of Kv4.2/4.3, the channels that produce the transient outward K^+^ current (I_to_), is reduced in atrial myocytes in mouse models of diet induced obesity and Type II DM. The mechanism for this reduction is unclear, but may involve impaired insulin signaling as Kv4.2 expression has also been shown to be decreased in a cardiac specific insulin receptor knockout mouse (Lopez-Izquierdo et al., 2014).

Other investigations have demonstrated that Type I diabetic mice (STZ treatment) display reduced expression of small conductance Ca^2+^-activated K^+^ channels (SK), including SK2 and SK3 (Yi et al., 2015). These changes in the expression of SK channels occurred in association with increased oxidative stress. Consistent with these animal studies, expression of SK channels (SK1, 2, and 3) was found to be reduced in the atria of human patients with chronic AF (Yu et al., 2012; Skibsbye et al., 2014). Additional studies are needed to better understand the links between SK channel function, diabetes, and AF.

#### Gap Junctions

**Figure 5:**
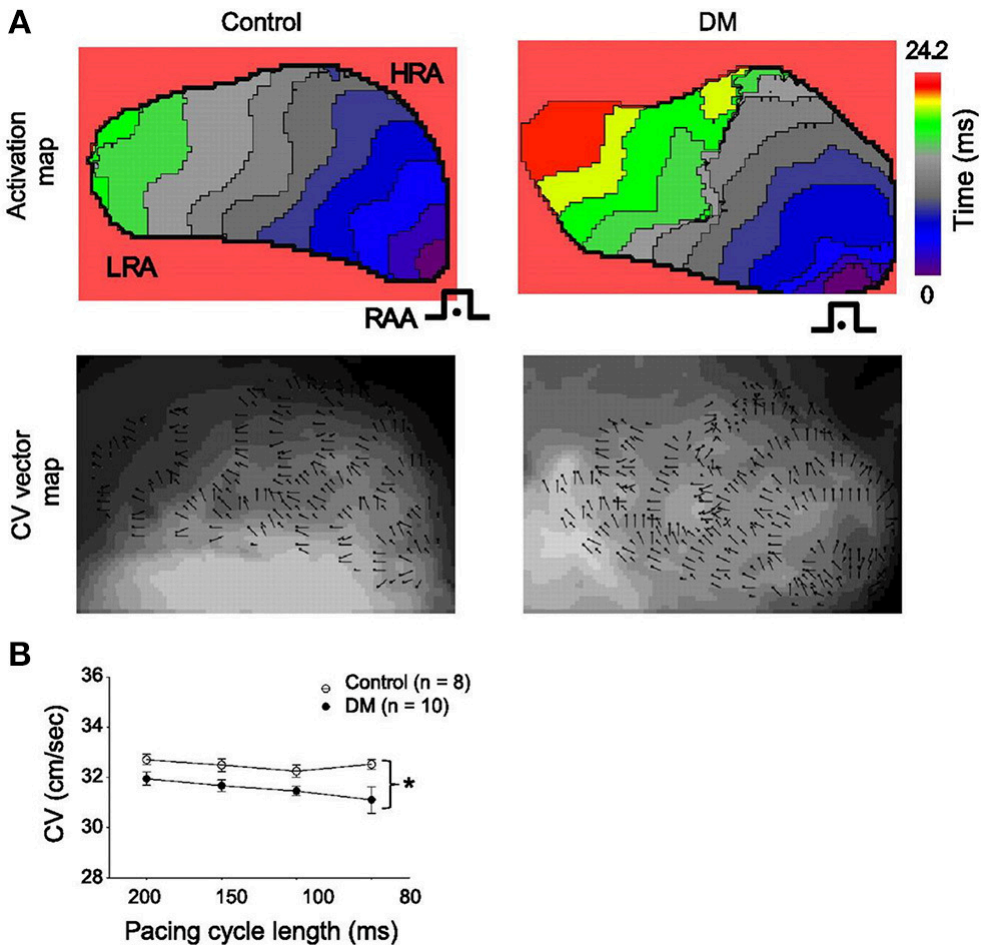
**(A)** representative activation maps (*top*) and conduction velocity (CV) vector maps (*bottom*) from control (*left*) and DM (*right*) rats. The isochrones on the activation maps are drawn at 2.2 ms intervals. Pacing is (•) from the right atrial appendage (RAA) at a cycle length of 200 ms. The color scale bar ranged from 0 to 24.2 ms. Isochronal crowding is evident within right atrium (RA) of DM rats. The CV vector map and the direction and magnitude of focal CV vectors varies widely within RA from DM rats. **(B)** Mean CVs at different pacing cycle lengths in control (*n* = 8) and DM (*n* = 10) rats. ^*^P < 0.01 vs. control. Reproduced with permission from Watanabe et al. (2012).

Gap junctions play an integral role in cell to cell communication and electrical conduction. These gap junctions are composed of connexin (Cx) proteins, with Cx40 and Cx43 being the predominant isoforms in the atrial myocardium. While data are limited, there is evidence of changes in Cx expression and function in the atria in DM. For example, it has been shown that Cx40 expression is reduced in the atria in a rat model of Type I DM. Consistent with this alteration in Cx40, optical mapping experiments showed that conduction velocity in the atria was reduced and conduction patterns lacked homogeneity (Figure 5) (Watanabe et al., 2012). These observations are in agreement with studies showing increased atrial arrhythmogenesis in Cx40 deficient mice (Hagendorff et al., 1999).

Cx43 expression in the atria does not appear to be changed in the setting of Type I or Type II DM. However, there is evidence that its subcelluar distribution is altered. For example, in Type II diabetic rats, Cx43 expression was not changed, but these animals displayed increased lateralization of Cx43 in the left atrium (Li et al., 2016). Lateralization of connexins would increase conduction heterogeneity because fewer Cx43-comprised gap junctions would be localized to the intercalated disks. Consistent with this hypothesis, lateralization of Cx43 and altered conduction has also been demonstrated in the ventricles of rats with Type I DM (Nygren et al., 2007).

### Translational Considerations: Glycemic Control and AF

A few clinical studies have attempted to elucidate association between markers of glycemic control and AF. An analysis of the Framingham Heart Study Offspring cohort followed for ≤10 years reported no association between insulin resistance and incident AF (Fontes et al., 2012). In contrast, in the Malmo Preventive Project, higher levels of fasting plasma insulin were associated with a lower risk of AF (Johnson et al., 2014). This relationship was significant only in the male cohort although the proportion of women included in this study was lower (33%). Furthermore, the association was weaker in subjects with elevated fasting glucose. Spontaneous hypoglycemia has been reported to be significantly associated with the development of AF in Type II DM (Ko et al., 2018). However, the mechanism underlying this relationship is unknown. Our experimental data in Type I DM demonstrates that acute and chronic insulin therapy prevents the downregulation of the sodium current and reduces the inducibility of AF. This effect is mediated, in part, via phosphatidylinositol 3,4,5-triphosphate (Bohne et al., 2018). We have also shown that insulin treatment in this model also reduces the development of atrial fibrosis. Together these data suggest that insulin mitigates, at least in part, the development of the substrate for atrial reentry (Krishnaswamy et al., 2015).

A Danish nationwide cohort study investigated the role of thiazlidinediones compared to other second line diabetes therapy in 108,624 patients with DM and without prior AF who were treated with metformin or sulfonylurea as first line drug therapy (Pallisgaard et al., 2017). Thiazlidinediones compared to other second line diabetes therapy significantly reduced the risk of AF (HR 0.76, 95% CI 0.57–1.00). A subsequent meta-analysis was undertaken including three randomized clinical trials and four observational studies of 130,854 diabetic patients (Zhang et al., 2017). Overall, thiazlidinediones were associated with a 27% lower risk of developing AF compared to controls. Pioglitazone was reported to have a more beneficial effect for AF prevention compared to rosiglitazone. In an experimental model of Type II DM, rosiglitazone attenuated the atrial structural remodeling and vulnerability to AF (Liu et al., 2014). A more recent study, the Bypass Angioplasty Revascularization Investigation 2 Diabetes trial, randomized patients with Type II DM to insulin sensitization therapy vs. insulin provision therapy. The incidence of AF was similar in in both patient groups (HR 0.91, 95% CI 0.60–1.38). Furthermore, the incidence rate of AF in those treated with thiazolidinediones was similar in those not treated with thiazolidinediones. Together these data suggest that glycemic control prevents adverse atrial remodeling and propensity to AF in DM. However, the current data do not support a unique drug specific effect for AF prevention.

### Vascular Disease in DM

DM is associated with an accelerated development and increased risk of atherosclerosis. However, DM has been reported to be an independent risk factor for AF in most observational studies (Xiong et al., 2018). Nevertheless, coronary microvascular dysfunction characterized by reduced coronary flow reserve and endothelial dysfunction are associated with DM and precede the development of overt cardiac disease (Kibel et al., 2017). These abnormalities may develop as a consequence of metabolic, hormonal, hemodynamic, neural, and other factors associated with DM. Abnormalities of endothelial function may precede the development of AF (Shaikh et al., 2016). However, whether the presence of microvascular dysfunction directly influences the substrate for AF requires more experimental and clinical investigation.

### Autonomic Dysfunction and AF

Abnormalities of the autonomic nervous system may play a role in the pathogenesis of AF. Autonomic neuropathy is a frequent complication associated with DM although data on the association of this complication with AF is limited. A strong relationship between autonomic dysfunction measured using heart rate variability as the marker of autonomic function and silent AF has been reported in patients with type 2 DM (Rizzo et al., 2015). Heart rate recovery, another index of autonomic function, has also been reported to be predictive of a risk for AF in type 2 DM independent of left atrial volume index and other clinical factors including hypertension and coronary artery disease suggesting a role for autonomic dysfunction in the pathogenesis of AF in DM (Negishi et al., 2013). The underlying cellular mechanisms have not been extensively studied and require further investigation.

### Catheter Ablation and AF

Catheter ablation is an established therapy for symptomatic patients with AF. Some (Anselmino et al., 2015; Bunch et al., 2015; Akkaya et al., 2018; Budzianowski et al., 2018) but not all (Bogossian et al., 2016; Winkle et al., 2016) studies have suggested that recurrence rates in patients with DM undergoing catheter ablation are higher than in non-diabetic patients. However, in a meta-analysis of 15 studies including 1,464 patients with DM, long term maintenance of sinus rhythm following a redo procedure was similar to rates reported among the general population undergoing catheter ablation. In this meta-analysis, higher body mass index and higher glycated hemoglobin levels were associated with higher AF recurrence rates suggesting a role for the metabolic abnormalities in DM in promoting arrhythmogenesis (Anselmino et al., 2015).

### Role of Computational Modeling

Computational biology and modeling can be powerful approaches for studying the mechanistic basis for cardiac arrhythmias, including in DM. Whenever possible, these approaches should be considered in order to complement clinical and basis science research as well as to generate new hypotheses that can then be tested in established models and in patients. Computational approaches have proven to be a valuable and important approach in assessing the links between DM and AF. In this regard, a recent study used a machine learning approach to automatically select studies for inclusion in an updated meta-analysis of the association between DM and new onset AF (Xiong et al., 2018). This study showed that machine learning could accurately and objectively select studies for inclusion in meta-analyses, which is important given that the exponential growth in numbers of publications makes manual selection increasingly challenging. This machine learning study demonstrates that DM is a strong, independent risk factor AF (Xiong et al., 2018).

Computational modeling and simulations based on mechanistic insights into AF triggers and substrate derived from both experimental and clinical studies may also facilitate the development of safer and more effective therapeutic interventions (Boyle et al., 2016; Grandi and Maleckar, 2016). In this regard, advanced analysis of the ECG and integration of detailed clinical data including cardiac imaging, biomarkers and genomics may facilitate the development of more precision directed medical therapy (Gillis et al., 2013; Boyle et al., 2016). Future studies are required to evaluate the role of personalized computational models for individualizing therapeutic strategies for the AF patient with DM.

### Future Research Directions

Experimental data demonstrate that significant atrial electrical, structural and autonomic remodeling occur in both Type I and Type II DM and increase the vulnerability to AF. Whether the insulin resistance in type II DM activates unique intracellular signaling pathways independent of obesity requires further investigation. Future studies are also required to investigate the inter-relationships between obesity, hypertension, DM, obstructive sleep apnea, and other factors associated with the metabolic syndrome including systemic inflammation and oxidative stress. The relationship between incident AF and glycemic control also requires further investigation. In addition, the role of computational models in directing more precision therapy for AF in this setting requires further study.

## Author Contributions

All authors listed have made a substantial, direct and intellectual contribution to the work, and approved it for publication.

### Conflict of Interest Statement

SW–Consulting Arca Biopharma and < 5,000. AG–Medtronic Inc research support. The remaining authors declare that the research was conducted in the absence of any commercial or financial relationships that could be construed as a potential conflict of interest.
